# The Convention of Teachers at Louisville

**Published:** 1859-04

**Authors:** 


### EDITORIAL DEPARTMENT.

*The Convention of Teachers at Louisville.*—We have no intention of writing an article on the fruitful subject of Medical Education; not that we do not think that the present system could not be improved, but because we doubt the utility of a discussion on this subject before the coming convention of Professors at Louisville. What will be the result of this meeting, it is impossible to say; but we have no idea that medical schools will ever be brought to consider themselves bound to any definite system, prescribed by the American Medical Association, when it will be for their interest to do otherwise. The American Medical Association is a body which changes its members every year; the places of its meeting being changed yearly to different parts of our immense country, the delegates are but few that attend regularly. This is an insuperable objection to a general plan to regulate the sessions or requirements of colleges, the faculties of which are permanent, composed of men of distinguished position, and who are animated by an intense spirit of rivalry in regard to the several institutions to which they are attached; a rivalry, sometimes, not so much in regard to the excellence of their system, as to the number of students which assemble to hear them lecture, and the number of graduates which they turn out. Too often is utility in the system of teaching sacrificed to popularity. We cannot believe that a school striving to make headway against a powerful rival will ever feel itself bound not to shorten its term of study, or be lenient in the matter of preliminary education, or even, we might add, in its requisites for graduation. They do not precisely desire to have the reputation of being *excessively* rigid in their final examinations, and can ease their consciences by the reflection that they present facilities for information, of which their students may take advantage if they desire.

Though we cannot hope that much will result from this movement, we are glad to see the trial made by the Association; and opine that the only true way is to bring the teachers together. It is desirable, then, that every school in the country should be represented, with definite instructions from the faculties of which they are members.

